# Supplementary material for: Estradiol Regulates the Expression and Secretion of Antimicrobial Peptide S100A7 via the ERK1/2-Signaling Pathway in Goat Mammary Epithelial Cells
Source: Animals (Basel). 2022 Nov 8;12(22):3077. doi: 10.3390/ani12223077 (PMC9687026; doi:10.3390/ani12223077)
Supplement: Supplementary file 1 [file animals-12-03077-s001.zip › animals-1993695-supplementary.pdf]

Supplementary material

1、The cell viability(OD) for figure 4A was showed in table S1.

Table S1. Cell viability(OD) of gMECs with LPS or estradiol treatment for 24 h

Replicates	Control	LPS(5 μg/mL)	1 nM E2	10 nM E2	100 nM E2
Replicate 1	0.381	0.381	0.385	0.386	0.393
Replicate 2	0.388	0.392	0.381	0.392	0.389
Replicate 3	0.381	0.377	0.408	0.383	0.395

2、The level of S100A7 mRNA expression for figure 4B was showed in table S2.

Table S2. The level of S100A7 mRNA expression of gMECs with LPS or estradiol treatment for 6 h

Replicates	Control	LPS(5 μg/mL)	1 nM E2	10 nM E2	100 nM E2	LPS+ 1nM E2	LPS+ 10 nM E2	LPS+ 100 nM E2
Replicate 1	0.978	2.123	0.746	1.729	2.096	2.138	2.739	2.727
Replicate 2	1.014	2.265	0.799	1.654	1.646	1.873	2.572	2.938
Replicate 3	0.829	1.982	0.988	1.805	1.871	2.404	2.907	3.148

3、**The concentration of S100A7 secretion in gMECs for figure 4C was showed in table S3.**

Table S3. The concentration of S100A7 secretion in gMECs with LPS or estradiol treatment for 6 h

Replicates	Control	LPS(5 μg/mL)	1 nM E2	10 nM E2	100 nM E2	LPS+ 1nM E2	LPS+ 10 nM E2	LPS+ 100 nM E2
Replicate 1	15.39	30.64	16.23	26.17	29.75	27.54	25.54	25.8
Replicate 2	17.27	22.25	17.43	26.96	27.46	24.74	24.3	28.23
Replicate 3	14.97	26.445	14.12	25.88	25.97	21.56	24.35	27.015

4、**The relative strip gray of protein with estradiol treatment in gMECs for figure 5B was showed in table S4.**

Group	Replicates	pERK/ERK	p-AKT/AKT	p-p38/p38	p-JNK/JNK
Control	Replicate 1	1	1	1	1
	Replicate 2	1	1	1	1
	Replicate 3	1	1	1	1
1 nM E2	Replicate 1	1.192	0.876	1.118	0.910
	Replicate 2	1.137	0.970	0.928	0.937
	Replicate 3	1.164	0.923	1.023	0.924
10 nM E2	Replicate 1	0.988	0.904	1.067	1.074
	Replicate 2	1.265	1.069	0.861	1.065
	Replicate 3	1.127	0.986	0.964	1.070
100 nM E2	Replicate 1	1.998	1.060	0.991	0.873
	Replicate 2	2.171	0.979	1.022	0.973
	Replicate 3	1.824	1.218	1.007	0.923

Table S4. The relative strip gray of protein with estradiol treatment for 6 h

5、**The level of S100A7 mRNA expression for figure 6A was showed in table S5.**

Table S5. The level of S100A7 mRNA expression in gMECs after treatment for 6 h

Replicates	Control	LPS(5 μg/mL)	0.1 μM E2	1 μM ICI	0.1 μM E2+ 1 μM ICI	0.1 μM G1	1 μM G15	0.1 μM E2+ 1 μM G15
Replicate 1	1.00	2.363	1.796	1.073	1.186	2.205	0.939	1.037
Replicate 2	1.284	2.265	1.646	1.111	1.170	2.537	0.791	1.336
Replicate 3	1.086	2.626	1.650	1.050	1.212	2.209	1.220	1.160

6、**The concentration of S100A7 secretion in gMECs for figure 6B was showed in table S6.**

Table S6. The concentration of S100A7 secretion in gMECs after treatment for 6 h

Replicates	Control	LPS(5 μg/mL)	0.1 μM E2	1 μM ICI	0.1 μM E2+ 1 μM ICI	0.1 μM G1	1 μM G15	0.1 μM E2+ 1 μM G15
Replicate 1	19.09	32.66	34.21	21.11	21.52	28.44	22.77	28.83
Replicate 2	20.12	32.86	33.21	20.47	29.10	27.88	21.06	20.10
Replicate 3	20.44	33.85	35.42	19.32	22.66	28.58	22.53	25.54
