# Supplementary figures and images for: Estradiol Regulates the Expression and Secretion of Antimicrobial Peptide S100A7 via the ERK1/2-Signaling Pathway in Goat Mammary Epithelial Cells
Source: Animals (Basel). 2022 Nov 8;12(22):3077. doi: 10.3390/ani12223077 (PMC9687026; doi:10.3390/ani12223077)

**Figure S1.** Original western blot figures for Figures 3F and 5A.

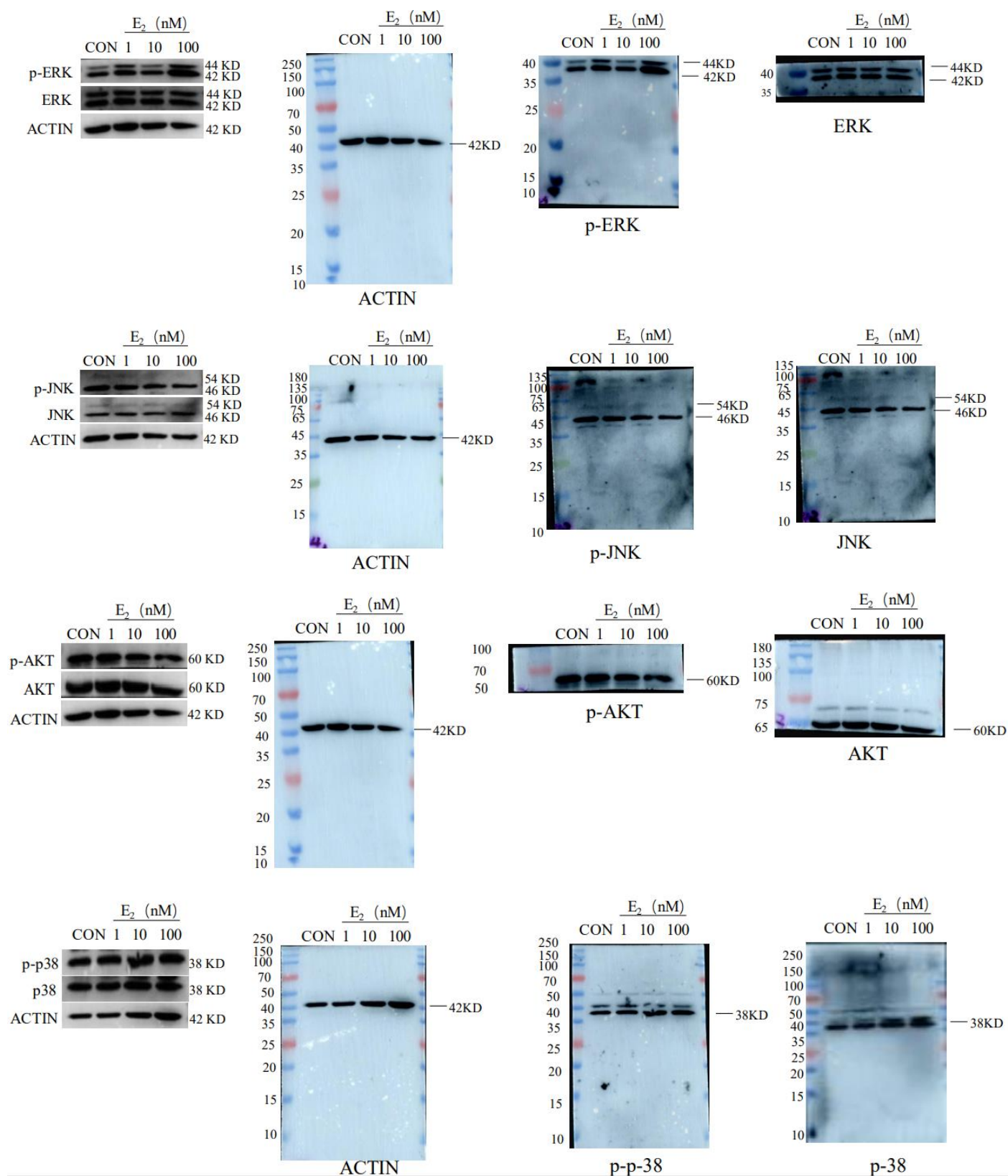

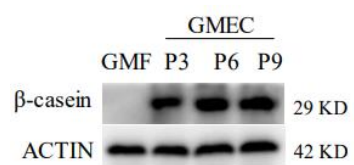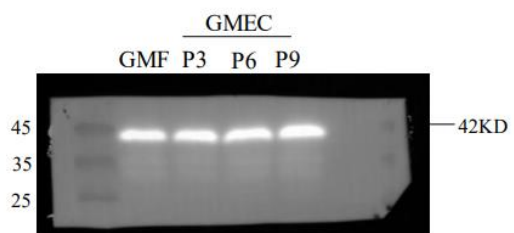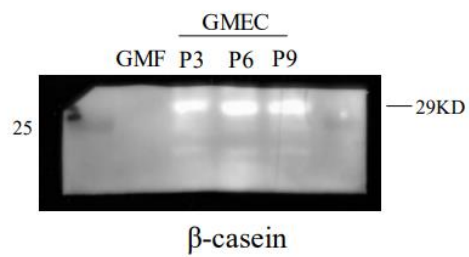

Supplement: Supplementary file 1 [file animals-12-03077-s001.zip › Figure S1. Original western blot figures for figure 3F&5A.pdf]
